# Di-μ-chlorido-bis­{[1,2-bis­(pyridin-2-ylmeth­oxy)benzene-κ^4^ *N*,*O*,*O*′,*N*′]chloridocadmium}

**DOI:** 10.1107/S1600536811037858

**Published:** 2011-09-30

**Authors:** Jin-Sheng Gao, Ying-Hui Yu, Ying Liu, Guang-Feng Hou

**Affiliations:** a Engineering Research Center of Pesticides of Heilongjiang University, Heilongjiang University, Harbin 150050, People’s Republic of China; b College of Chemistry and Materials Science, Heilongjiang University, Harbin 150080, People’s Republic of China

## Abstract

In centrosymmetric dinuclear title compound, [Cd_2_Cl_4_(C_18_H_16_N_2_O_2_)_2_], the Cd^II^ atom is seven-coordinated in a penta­gonal–bipyramidal environment defined by two N atoms and two O atoms from one ligand and three Cl^−^ anions, two of which are bridging. A π–π inter­action between adjacent pyridine rings [centroid–centroid distance = 3.773 (1) Å] further stablizes the dimer.

## Related literature

For general background to flexible bipyridyl-based ligands, see: Wang *et al.* (2004) ▶; Oh *et al.* (2005) ▶. For the synthesis of the ligand, see: Liu *et al.* (2010*a* ▶,*b* ▶). For a related structure, see: Liu *et al.* (2011 ▶).
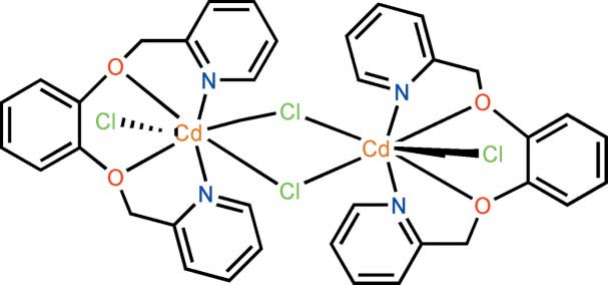


## Experimental

### 

#### Crystal data

[Cd_2_Cl_4_(C_18_H_16_N_2_O_2_)_2_]
*M* *_r_* = 951.26
Monoclinic, 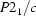

*a* = 10.833 (2) Å
*b* = 10.968 (2) Å
*c* = 16.095 (3) Å
β = 109.14 (3)°
*V* = 1806.6 (6) Å^3^
*Z* = 2
Mo *K*α radiation
μ = 1.52 mm^−1^
*T* = 293 K
0.23 × 0.22 × 0.20 mm

#### Data collection

Rigaku R-AXIS RAPID diffractometer
Absorption correction: multi-scan (*ABSCOR*; Higashi, 1995 ▶) *T*_min_ = 0.722, *T*_max_ = 0.751
17295 measured reflections
4128 independent reflections
3686 reflections with *I* > 2σ(*I*)
*R*_int_ = 0.021

#### Refinement

*R*[*F* ^2^ > 2σ(*F* ^2^)] = 0.019
*wR*(*F* ^2^) = 0.048
*S* = 1.05
4128 reflections
226 parameters
H-atom parameters constrained
Δρ_max_ = 0.36 e Å^−3^
Δρ_min_ = −0.24 e Å^−3^

### 

Data collection: *RAPID-AUTO* (Rigaku, 1998 ▶); cell refinement: *RAPID-AUTO*; data reduction: *CrystalClear* (Rigaku/MSC, 2002 ▶); program(s) used to solve structure: *SHELXS97* (Sheldrick, 2008 ▶); program(s) used to refine structure: *SHELXL97* (Sheldrick, 2008 ▶); molecular graphics: *SHELXTL* (Sheldrick, 2008 ▶); software used to prepare material for publication: *SHELXL97*.

## Supplementary Material

Crystal structure: contains datablock(s) I, global. DOI: 10.1107/S1600536811037858/ng5223sup1.cif

Structure factors: contains datablock(s) I. DOI: 10.1107/S1600536811037858/ng5223Isup2.hkl

Additional supplementary materials:  crystallographic information; 3D view; checkCIF report

## Figures and Tables

**Table 1:** Selected bond lengths (Å)

Cd1—N2	2.3920 (15)
Cd1—N1	2.3958 (15)
Cd1—Cl2	2.5103 (6)
Cd1—O1	2.6197 (14)
Cd1—Cl1	2.6197 (6)
Cd1—O2	2.6288 (14)
Cd1—Cl1^i^	2.6873 (11)

Symmetry code: (i) 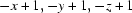.

**Fig. 1.**
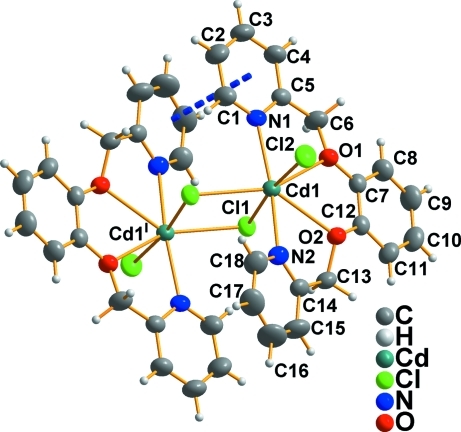
The molecular structure of the title compound, showing displacement ellipsoids at the 50% probability level for non-H atoms. Symmetry codes: (I) -x+1,-y+1,1-z.

## supplementary crystallographic information

### Comment

Aromatic molecules containing two pyridyl groups have been widely used as building blocks for new supramolecular architectures in recent years. Compared with rigid bridging ligands, flexible bipyridine ligands are able to generate some unusual frameworks (Wang *et al.*, 2004; Oh *et al.*, 2005). In continuation of previous works (Liu *et al.*, 2010*a*; 2010*b*), we report the crystal structure of the title compound.

In centrosymmetric dinuclear [CdCl2(C18H16N2O2)]2, the CdII atom is seven-coordinated in a pentagonal bipyramidal environment defined by two N atoms and two O atoms from one ligand and three chlorides. Two of chlorides serve are bridging. A π—π interaction between adjacent pyridine rings [center to center distance 3.773?(1)?Å] further stablizes the dimer (Fig. 1, Table 1).

### Experimental

The 1,2-bis(pyridin-2-ylmethoxy)benzene ligand was synthesized according to a literature method (Liu *et al.*,2010*a*). A solution of CdCl_2_^.^5H_2_O (0.2 mmol,0.0456 g) in water (2 ml) was added to a solution of the ligand (0.2 mmol, 0.0584 g) in 5 ml methanol under constant stirring; the solution was stirred for about 1 hour and then filted. The filtate was maintained for about one week under room temperature to give colorless block-like crystals.

### Refinement

H atoms bound to C atoms were placed in calculated positions and treated as riding on their parent atoms, with C—H = 0.93 Å (aromatic); C—H = 0.97 Å (methylene), and with *U*_iso_(H) = 1.2*U*_eq_(C).

### Crystal data

**Table:**

[Cd_2_Cl_4_(C_18_H_16_N_2_O_2_)_2_]	*F*(000) = 944
*M**_r_* = 951.26	*D*_x_ = 1.749 Mg m^−^^3^
Monoclinic, *P*2_1_/*c*	Mo *K*α radiation, λ = 0.71073 Å
Hall symbol: -P 2ybc	Cell parameters from 15418 reflections
*a* = 10.833 (2) Å	θ = 3.3–27.5°
*b* = 10.968 (2) Å	µ = 1.52 mm^−^^1^
*c* = 16.095 (3) Å	*T* = 293 K
β = 109.14 (3)°	Bolck, colorless
*V* = 1806.6 (6) Å^3^	0.23 × 0.22 × 0.20 mm
*Z* = 2	

### Data collection

**Table:**

Rigaku R-AXIS RAPID diffractometer	4128 independent reflections
Radiation source: fine-focus sealed tube	3686 reflections with *I* > 2σ(*I*)
graphite	*R*_int_ = 0.021
ω scans	θ_max_ = 27.5°, θ_min_ = 3.3°
Absorption correction: multi-scan (*ABSCOR*; Higashi, 1995)	*h* = −14→13
*T*_min_ = 0.722, *T*_max_ = 0.751	*k* = −14→14
17295 measured reflections	*l* = −20→20

### Refinement

**Table:**

Refinement on *F*^2^	Primary atom site location: structure-invariant direct methods
Least-squares matrix: full	Secondary atom site location: difference Fourier map
*R*[*F*^2^ > 2σ(*F*^2^)] = 0.019	Hydrogen site location: inferred from neighbouring sites
*wR*(*F*^2^) = 0.048	H-atom parameters constrained
*S* = 1.05	*w* = 1/[σ^2^(*F*_o_^2^) + (0.0229*P*)^2^ + 0.5495*P*] where *P* = (*F*_o_^2^ + 2*F*_c_^2^)/3
4128 reflections	(Δ/σ)_max_ = 0.002
226 parameters	Δρ_max_ = 0.36 e Å^−^^3^
0 restraints	Δρ_min_ = −0.24 e Å^−^^3^

### Special details

**Table:**

Geometry. All esds (except the esd in the dihedral angle between two l.s. planes) are estimated using the full covariance matrix. The cell esds are taken into account individually in the estimation of esds in distances, angles and torsion angles; correlations between esds in cell parameters are only used when they are defined by crystal symmetry. An approximate (isotropic) treatment of cell esds is used for estimating esds involving l.s. planes.
Refinement. Refinement of F^2^ against ALL reflections. The weighted R-factor wR and goodness of fit S are based on F^2^, conventional R-factors R are based on F, with F set to zero for negative F^2^. The threshold expression of F^2^ > 2sigma(F^2^) is used only for calculating R-factors(gt) etc. and is not relevant to the choice of reflections for refinement. R-factors based on F^2^ are statistically about twice as large as those based on F, and R- factors based on ALL data will be even larger.

### Fractional atomic coordinates and isotropic or equivalent isotropic displacement parameters (Å^2^)

**Table:**

	*x*	*y*	*z*	*U*_iso_*/*U*_eq_	
C1	0.6708 (2)	0.4345 (2)	0.34515 (14)	0.0565 (5)	
H1	0.5939	0.4787	0.3220	0.068*	
C2	0.7136 (2)	0.3659 (3)	0.28896 (15)	0.0679 (7)	
H2	0.6668	0.3642	0.2291	0.081*	
C3	0.8267 (2)	0.2998 (2)	0.32244 (16)	0.0660 (6)	
H3	0.8573	0.2515	0.2859	0.079*	
C4	0.8935 (2)	0.3064 (2)	0.41076 (15)	0.0546 (5)	
H4	0.9708	0.2631	0.4350	0.065*	
C5	0.84454 (17)	0.37838 (17)	0.46357 (13)	0.0406 (4)	
C6	0.9171 (2)	0.3807 (2)	0.55999 (13)	0.0516 (5)	
H6A	0.8960	0.3081	0.5870	0.062*	
H6B	1.0103	0.3801	0.5696	0.062*	
C7	0.93736 (16)	0.48931 (16)	0.69088 (11)	0.0376 (4)	
C8	1.0417 (2)	0.41839 (18)	0.73965 (14)	0.0507 (5)	
H8	1.0810	0.3649	0.7111	0.061*	
C9	1.0880 (2)	0.4263 (2)	0.83053 (15)	0.0565 (5)	
H9	1.1587	0.3788	0.8628	0.068*	
C10	1.0299 (2)	0.5037 (2)	0.87260 (13)	0.0554 (5)	
H10	1.0605	0.5081	0.9337	0.066*	
C11	0.9252 (2)	0.57610 (18)	0.82491 (13)	0.0473 (4)	
H11	0.8860	0.6286	0.8542	0.057*	
C12	0.87928 (17)	0.57023 (15)	0.73393 (12)	0.0367 (4)	
C13	0.68776 (19)	0.68425 (18)	0.72191 (12)	0.0440 (4)	
H13A	0.7328	0.7322	0.7736	0.053*	
H13B	0.6476	0.6149	0.7404	0.053*	
C14	0.58438 (17)	0.76039 (16)	0.65859 (12)	0.0417 (4)	
C15	0.5092 (3)	0.8376 (2)	0.69083 (17)	0.0681 (7)	
H15	0.5224	0.8415	0.7508	0.082*	
C16	0.4150 (3)	0.9084 (2)	0.6327 (2)	0.0826 (9)	
H16	0.3629	0.9600	0.6529	0.099*	
C17	0.3989 (2)	0.9021 (2)	0.5454 (2)	0.0685 (7)	
H17	0.3379	0.9513	0.5052	0.082*	
C18	0.4747 (2)	0.82148 (18)	0.51789 (16)	0.0546 (5)	
H18	0.4632	0.8167	0.4581	0.066*	
Cd1	0.660311 (11)	0.587855 (10)	0.515336 (7)	0.03233 (5)	
Cl1	0.57974 (4)	0.42461 (4)	0.60476 (3)	0.03852 (9)	
Cl2	0.76064 (5)	0.75247 (5)	0.44987 (3)	0.05070 (12)	
N1	0.73405 (15)	0.44122 (14)	0.43174 (10)	0.0401 (3)	
N2	0.56444 (15)	0.74944 (13)	0.57321 (10)	0.0410 (3)	
O1	0.88495 (11)	0.48583 (11)	0.60035 (8)	0.0393 (3)	
O2	0.77949 (11)	0.64229 (11)	0.68148 (8)	0.0375 (3)	

### Atomic displacement parameters (Å^2^)

**Table:**

	*U*^11^	*U*^22^	*U*^33^	*U*^12^	*U*^13^	*U*^23^
C1	0.0481 (11)	0.0767 (15)	0.0439 (11)	0.0117 (11)	0.0142 (9)	−0.0047 (10)
C2	0.0637 (14)	0.0959 (18)	0.0434 (12)	0.0060 (14)	0.0166 (10)	−0.0174 (12)
C3	0.0620 (13)	0.0842 (17)	0.0582 (13)	0.0049 (13)	0.0282 (11)	−0.0254 (12)
C4	0.0476 (11)	0.0610 (12)	0.0578 (12)	0.0089 (10)	0.0210 (10)	−0.0143 (10)
C5	0.0395 (9)	0.0410 (9)	0.0452 (10)	−0.0007 (8)	0.0190 (8)	−0.0059 (8)
C6	0.0551 (12)	0.0526 (11)	0.0460 (11)	0.0186 (10)	0.0150 (9)	−0.0048 (9)
C7	0.0344 (8)	0.0401 (9)	0.0367 (9)	−0.0027 (7)	0.0097 (7)	0.0005 (7)
C8	0.0434 (10)	0.0517 (11)	0.0524 (11)	0.0103 (9)	0.0095 (9)	0.0008 (9)
C9	0.0483 (11)	0.0593 (13)	0.0507 (12)	0.0091 (10)	0.0012 (9)	0.0092 (10)
C10	0.0581 (12)	0.0618 (13)	0.0368 (10)	−0.0025 (11)	0.0027 (9)	0.0034 (9)
C11	0.0530 (11)	0.0480 (10)	0.0374 (9)	−0.0011 (9)	0.0099 (8)	−0.0043 (8)
C12	0.0348 (8)	0.0355 (8)	0.0373 (9)	−0.0031 (7)	0.0085 (7)	0.0007 (7)
C13	0.0471 (10)	0.0507 (10)	0.0361 (9)	0.0023 (9)	0.0160 (8)	−0.0081 (8)
C14	0.0413 (9)	0.0377 (9)	0.0487 (10)	−0.0033 (8)	0.0184 (8)	−0.0117 (8)
C15	0.0770 (16)	0.0662 (14)	0.0667 (15)	0.0145 (13)	0.0311 (13)	−0.0216 (12)
C16	0.0782 (18)	0.0681 (16)	0.104 (2)	0.0297 (14)	0.0327 (17)	−0.0219 (15)
C17	0.0594 (14)	0.0456 (12)	0.0910 (19)	0.0189 (11)	0.0119 (13)	−0.0043 (12)
C18	0.0595 (12)	0.0414 (10)	0.0585 (12)	0.0116 (10)	0.0132 (10)	0.0016 (9)
Cd1	0.03316 (7)	0.03389 (7)	0.03234 (7)	0.00016 (5)	0.01399 (5)	0.00062 (5)
Cl1	0.0354 (2)	0.0422 (2)	0.0366 (2)	−0.00347 (17)	0.00993 (16)	0.00500 (17)
Cl2	0.0531 (3)	0.0566 (3)	0.0449 (2)	−0.0115 (2)	0.0195 (2)	0.0106 (2)
N1	0.0396 (8)	0.0453 (8)	0.0383 (8)	0.0041 (7)	0.0168 (6)	−0.0027 (6)
N2	0.0439 (8)	0.0360 (7)	0.0436 (8)	0.0059 (7)	0.0148 (7)	−0.0010 (6)
O1	0.0369 (6)	0.0421 (6)	0.0380 (6)	0.0080 (5)	0.0109 (5)	−0.0019 (5)
O2	0.0389 (6)	0.0393 (6)	0.0345 (6)	0.0039 (5)	0.0124 (5)	−0.0020 (5)

### Geometric parameters (Å, °)

**Table:**

C1—N1	1.338 (3)	C11—H11	0.9300
C1—C2	1.368 (3)	C12—O2	1.381 (2)
C1—H1	0.9300	C13—O2	1.431 (2)
C2—C3	1.373 (3)	C13—C14	1.497 (3)
C2—H2	0.9300	C13—H13A	0.9700
C3—C4	1.369 (3)	C13—H13B	0.9700
C3—H3	0.9300	C14—N2	1.325 (2)
C4—C5	1.387 (3)	C14—C15	1.388 (3)
C4—H4	0.9300	C15—C16	1.376 (4)
C5—N1	1.330 (2)	C15—H15	0.9300
C5—C6	1.492 (3)	C16—C17	1.360 (4)
C6—O1	1.422 (2)	C16—H16	0.9300
C6—H6A	0.9700	C17—C18	1.376 (3)
C6—H6B	0.9700	C17—H17	0.9300
C7—O1	1.380 (2)	C18—N2	1.339 (3)
C7—C8	1.385 (3)	C18—H18	0.9300
C7—C12	1.396 (2)	Cd1—N2	2.3920 (15)
C8—C9	1.385 (3)	Cd1—N1	2.3958 (15)
C8—H8	0.9300	Cd1—Cl2	2.5103 (6)
C9—C10	1.362 (3)	Cd1—O1	2.6197 (14)
C9—H9	0.9300	Cd1—Cl1	2.6197 (6)
C10—C11	1.391 (3)	Cd1—O2	2.6288 (14)
C10—H10	0.9300	Cd1—Cl1^i^	2.6873 (11)
C11—C12	1.385 (3)	Cl1—Cd1^i^	2.6873 (11)
			
N1—C1—C2	123.4 (2)	C15—C14—C13	119.04 (19)
N1—C1—H1	118.3	C16—C15—C14	119.0 (2)
C2—C1—H1	118.3	C16—C15—H15	120.5
C1—C2—C3	118.9 (2)	C14—C15—H15	120.5
C1—C2—H2	120.6	C17—C16—C15	119.3 (2)
C3—C2—H2	120.6	C17—C16—H16	120.3
C4—C3—C2	118.7 (2)	C15—C16—H16	120.3
C4—C3—H3	120.7	C16—C17—C18	118.5 (2)
C2—C3—H3	120.7	C16—C17—H17	120.8
C3—C4—C5	119.2 (2)	C18—C17—H17	120.8
C3—C4—H4	120.4	N2—C18—C17	123.0 (2)
C5—C4—H4	120.4	N2—C18—H18	118.5
N1—C5—C4	122.40 (18)	C17—C18—H18	118.5
N1—C5—C6	119.51 (16)	N2—Cd1—N1	169.53 (5)
C4—C5—C6	118.06 (17)	N2—Cd1—Cl2	86.19 (4)
O1—C6—C5	111.39 (16)	N1—Cd1—Cl2	88.69 (4)
O1—C6—H6A	109.3	N2—Cd1—O1	124.10 (5)
C5—C6—H6A	109.3	N1—Cd1—O1	65.35 (5)
O1—C6—H6B	109.3	Cl2—Cd1—O1	94.05 (3)
C5—C6—H6B	109.3	N2—Cd1—Cl1	91.55 (4)
H6A—C6—H6B	108.0	N1—Cd1—Cl1	94.72 (4)
O1—C7—C8	124.05 (17)	Cl2—Cd1—Cl1	171.978 (16)
O1—C7—C12	116.44 (15)	O1—Cd1—Cl1	80.84 (3)
C8—C7—C12	119.51 (17)	N2—Cd1—O2	64.32 (5)
C7—C8—C9	120.6 (2)	N1—Cd1—O2	125.51 (5)
C7—C8—H8	119.7	Cl2—Cd1—O2	97.34 (3)
C9—C8—H8	119.7	O1—Cd1—O2	60.22 (4)
C10—C9—C8	119.8 (2)	Cl1—Cd1—O2	74.77 (3)
C10—C9—H9	120.1	N2—Cd1—Cl1^i^	82.91 (4)
C8—C9—H9	120.1	N1—Cd1—Cl1^i^	89.09 (4)
C9—C10—C11	120.51 (19)	Cl2—Cd1—Cl1^i^	100.64 (2)
C9—C10—H10	119.7	O1—Cd1—Cl1^i^	150.24 (3)
C11—C10—H10	119.7	Cl1—Cd1—Cl1^i^	86.69 (2)
C12—C11—C10	120.13 (19)	O2—Cd1—Cl1^i^	141.26 (3)
C12—C11—H11	119.9	Cd1—Cl1—Cd1^i^	93.31 (2)
C10—C11—H11	119.9	C5—N1—C1	117.47 (16)
O2—C12—C11	123.89 (16)	C5—N1—Cd1	123.73 (12)
O2—C12—C7	116.75 (15)	C1—N1—Cd1	118.10 (13)
C11—C12—C7	119.34 (17)	C14—N2—C18	118.17 (17)
O2—C13—C14	110.24 (15)	C14—N2—Cd1	121.82 (12)
O2—C13—H13A	109.6	C18—N2—Cd1	119.42 (13)
C14—C13—H13A	109.6	C7—O1—C6	115.30 (14)
O2—C13—H13B	109.6	C7—O1—Cd1	121.99 (10)
C14—C13—H13B	109.6	C6—O1—Cd1	115.63 (11)
H13A—C13—H13B	108.1	C12—O2—C13	115.29 (13)
N2—C14—C15	121.80 (19)	C12—O2—Cd1	121.12 (10)
N2—C14—C13	119.12 (15)	C13—O2—Cd1	110.67 (10)

Symmetry codes: (i) −*x*+1, −*y*+1, −*z*+1.
